# Culture-negative periprosthetic joint infection: an umbrella review of prevalence, diagnostic strategies, treatment, and methodological limitations

**DOI:** 10.5194/jbji-11-479-2026

**Published:** 2026-08-03

**Authors:** Guido Bocchino, Giulio Maccauro, Andrea Zampoli, Rocco Papalia, Pier Francesco Indelli, Daniel Pérez-Prieto, Javad Parvizi

**Affiliations:** 1 Department of Orthopedics and Geriatric Sciences, Catholic University of the Sacred Heart, Largo Francesco Vito, 8, 00168, Rome, Italy; 2 Department of Orthopedics, Ageing and Rheumatological Sciences, Fondazione Policlinico Universitario A. Gemelli IRCCS, Largo Agostino Gemelli 8, 00168, Rome, Italy; 3 Institute of Biomechanics, Paracelsus Medical University (PMU), Salzburg, Austria; 4 Department of Orthopaedic and Trauma Surgery, Università Campus Bio-Medico, Rome, Italy; 5 Research Unit of Orthopaedic and Trauma Surgery, Fondazione Policlinico Universitario Campus Bio-Medico, Rome, Italy; 6 The Breyer Center for Overseas Studies, Stanford University in Florence, Florence, Italy; 7 University of Trento, School of Medicine, Trento, Italy; 8 Orthopedic Department, Septic Unit, Hospital del Mar, Universitat Pompeu Fabra, Barcelona, Spain; 9 International Joint Center, Acibadem University, Istanbul, Türkiye

## Abstract

**Purpose**: Culture-negative periprosthetic joint infection (CN-PJI) poses a significant clinical challenge because antimicrobial selection relies on guesswork rather than robust clinical data. This umbrella review summarizes the prevalence of CN-PJI, diagnostic strategies (including molecular assays), treatment approaches, and outcomes. **Methods**: We conducted an umbrella review of published systematic reviews and meta-analyses on CN-PJI in adult hip and/or knee arthroplasties. Searches of major bibliographic databases were conducted in December 2025; two reviewers screened studies and extracted data on prevalence, reported risk factors, diagnostic criteria, use of molecular tests, specimen matrix (synovial fluid, periprosthetic tissue, and/or sonication fluid), surgical strategy, antimicrobial selection, and clinical outcomes (infection control or failure). Methodological quality was assessed with AMSTAR 2. **Results**: Nine reviews were included. Reported CN-PJI prevalence varied widely (11.0 %–63.6 %), reflecting substantial heterogeneity in study populations, diagnostic definitions, microbiological workflows, and pre-analytical factors. Prior antibiotic exposure before sampling was repeatedly identified as an important contributor to culture negativity. Molecular diagnostics, predominantly NGS (next-generation sequencing)-based approaches, were evaluated in five out of nine reviews, with highly variable organism detection rates depending on assay type, specimen source, and study context. Where meta-analytic estimates were available, pooled sensitivity ranged from 0.81 to 0.93, and pooled specificity ranged from 0.92 to 0.97. Treatment strategies and outcome definitions were inconsistently reported across reviews; when extractable, two-stage exchange was the most frequently described surgical strategy. **Conclusions**: CN-PJI remains common and variable across settings. Molecular diagnostics improve pathogen detection but do not eliminate false negatives. Reported failure rates varied across reviews and appeared to be clinically relevant, although differences in outcome definitions, treatment protocols, and follow-up duration limited direct comparison.

## 1 Introduction

Periprosthetic joint infection (PJI) remains one of the most serious complications following total joint arthroplasty (TJA), leading to repeated operations, prolonged antimicrobial exposure, functional decline, and substantial healthcare costs (Parvizi et al., 2018). Diagnostic frameworks have improved standardization, but they have also highlighted a persistent and clinically consequential subset of cases in which conventional cultures fail to identify the causative organism (Parvizi et al., 2018; McNally et al., 2021). Culture-negative periprosthetic joint infection (CN-PJI) is typically defined as an infection that meets accepted diagnostic criteria yet yields negative synovial and/or periprosthetic tissue cultures under routine laboratory conditions. The reported proportion of CN cases varies across cohorts and healthcare systems, reflecting heterogeneity in case mix, pre-analytic factors (e.g., antibiotic exposure), sampling techniques, the number and types of specimens obtained, culture media, and incubation durations. Prior antimicrobial therapy is consistently associated with lower culture yield, supporting the long-standing stewardship principle that, when clinically feasible, antibiotics should be withheld until appropriate diagnostic specimens are obtained (Osmon et al., 2013; Malekzadeh et al., 2010). Culture negativity is not a benign finding as CN-PJI is associated with meaningful failure rates (Li et al., 2023). Many initially culture-negative failures later become culture-positive, suggesting missed microbiological diagnoses rather than true pathogen-absent infections (Tan et al., 2018). Some strategies, such as implant sonication and optimizing conventional microbiology (e.g., transporting samples in blood culture bottles, obtaining multiple tissue samples, and prolonging the incubation period), have been introduced to improve microbial recovery and reduce the rate of CN-PJI (Trampuz et al., 2007; Rothenberg et al., 2017; Peel et al., 2016; Peel et al., 2017). Concurrently, the “molecular era” has introduced culture-independent diagnostics, including targeted PCR approaches (e.g., 16S rDNA PCR, multiplex PCR) and next-generation sequencing (NGS), particularly metagenomic NGS (mNGS). Early clinical studies suggested that NGS may identify organisms in a substantial proportion of cases that remain culture-negative, raising expectations that culture negativity could become a diminishing problem (Tarabichi et al., 2018; Goswami et al., 2022). More recently, systematic reviews and meta-analyses have evaluated the diagnostic performance of these molecular techniques, generally reporting higher sensitivity and diagnostic accuracy for NGS-based methods than for culture while also highlighting important limitations, including contamination risk, challenges in interpreting low-biomass signals, and incomplete standardization (Su et al., 2024; Olearo et al., 2025; Wang et al., 2025). At the same time, reviews of culture-negative PJI have reported heterogeneous, sometimes conflicting findings regarding prevalence, management strategies, and outcomes compared with culture-positive PJI (Lai et al., 2024; Li et al., 2023). Given these inconsistencies and the increasing number of systematic reviews addressing either CN-PJI specifically or diagnostic strategies relevant to CN-PJI, we conducted an umbrella review to summarize review-level evidence, identify areas of convergence and disagreement, and highlight major methodological limitations in the current literature, including heterogeneity and overlap among primary studies.

## 2 Materials and methods

This study was designed as an umbrella review to synthesize evidence from published systematic reviews and meta-analyses evaluating CN-PJI in the context of modern diagnostic strategies, including molecular diagnostics. The review was conducted and reported in accordance with the Preferred Reporting Items for Systematic Reviews and Meta-Analyses (PRISMA 2020) statement and methodological guidelines for umbrella reviews. Before starting the review, the International Prospective Register of Systematic Reviews (PROSPERO) was searched to identify ongoing or completed umbrella reviews on CN-PJI; however, this umbrella review was not registered prospectively.

### 2.1 Eligibility criteria

Eligible articles included systematic reviews and meta-analyses addressing CN-PJI in adult patients undergoing total joint arthroplasty.

The inclusion criteria were as follows:

1. systematic reviews and/or meta-analyses (with or without quantitative pooling);
2. reviews investigating CN-PJI, including epidemiology, diagnostic strategies, treatment approaches, or clinical outcomes;
3. studies including adult patients undergoing hip and/or knee arthroplasties;
4. articles published in peer-reviewed journals;
5. articles published in the English language.

The exclusion criteria were as follows:

1. primary clinical studies (randomized controlled trials, cohort studies, case–control studies, case series, or case reports)
2. narrative reviews without a reproducible systematic search methodology
3. reviews focusing exclusively on native joint septic arthritis
4. reviews that pooled PJI data without specific reference to culture-negative infections
5. non-English publications.

### 2.2 Search strategy

A comprehensive electronic literature search was conducted in December 2025 using MEDLINE via PubMed, Embase, the Cochrane Database of Systematic Reviews, and Web of Science. The search strategy combined controlled vocabulary terms, when available, and free-text terms related to periprosthetic joint infection, culture-negative infection, and review methodology. The core concepts included terms for periprosthetic or prosthetic joint infection, culture-negative or negative-culture infection, and systematic reviews or meta-analyses. Search strategies were adapted to the syntax and indexing structure of each database. No additional date restriction was applied. The search was restricted to articles published in English.

The complete search strategy for each database is provided in Table S1 in the Supplement. Reference lists of included reviews were also manually screened to identify additional eligible articles. The search was considered to be complete when no further eligible reviews were identified after iterative refinement of the search terms and manual reference checking.

### 2.3 Study selection

All retrieved records were imported into reference management software, and duplicates were removed. Two reviewers (Guido Bocchino and Pier Francesco Indelli) independently screened titles and abstracts for eligibility. A full-text review was then conducted to identify potentially eligible articles. Any disagreements were resolved through discussion and consensus. In the PRISMA flow diagram, reports categorized as “not retrieved” are records identified for full-text assessment for which the full text could not be obtained despite institutional and manual search efforts; these reports were therefore not assessed for eligibility and were not included in the synthesis.

### 2.4 Data extraction

Two reviewers (Giulio Maccauro and Daniel Pérez-Prieto) independently extracted data using a predefined data extraction form. The following information was collected from each included review: author and year of publication, journal and type of review (systematic review or meta-analysis), number and type of primary studies included; arthroplasty joint(s) studied (hip, knee, or mixed), definition of CN-PJI used; diagnostic methods evaluated (standard cultures, sonication, molecular diagnostics), reported surgical and antimicrobial treatment strategies, and reported clinical outcomes (infection control, failure, reoperation, complications). Because several included reviews addressed overlapping clinical questions and may have included overlapping primary studies, extracted data were summarized at the review level. Discrepancies in data extraction were resolved by consensus. The methodological quality of the included systematic reviews and meta-analyses was assessed using AMSTAR 2 (A Measurement Tool to Assess Systematic Reviews 2). Two reviewers (Rocco Papalia and Javad Parvizi) independently applied AMSTAR 2 to each included review; disagreements were resolved through discussion and consensus. In accordance with AMSTAR 2 guidance, no numerical score was calculated; instead, each review was assigned an overall confidence rating (high, moderate, low, or critically low). AMSTAR 2 ratings were used to contextualize and interpret the strength of the synthesized evidence and were not used as an exclusion criterion. Because umbrella reviews are vulnerable to overlap among primary studies included in different reviews, overlap was assessed using a citation matrix and the corrected covered area (CCA). For each included review, primary studies were extracted and entered into a citation matrix, with primary studies as rows and included reviews as columns. The CCA was calculated using the formula CCA = ( N-r)/(rc-r) , where N is the total number of study occurrences across reviews, r is the number of unique primary studies, and c is the number of included reviews. The degree of overlap was interpreted as slight (0 %–5 %), moderate (6 %–10 %), high (11 %–15 %), or very high ( > 15 %). The citation matrix, CCA calculation, and AMSTAR 2 overall confidence ratings are provided in Table S2. Given the observed overlap, we did not treat the cumulative numbers of primary studies or patients reported across reviews as independent pooled datasets. Data were synthesized primarily at the review level. Where reviews reported similar outcomes, findings were compared narratively rather than re-pooled across reviews. This approach was chosen to reduce the risk of double-counting and overinterpretation given likely overlap among primary studies.

## 3 Results

**Figure 1:**
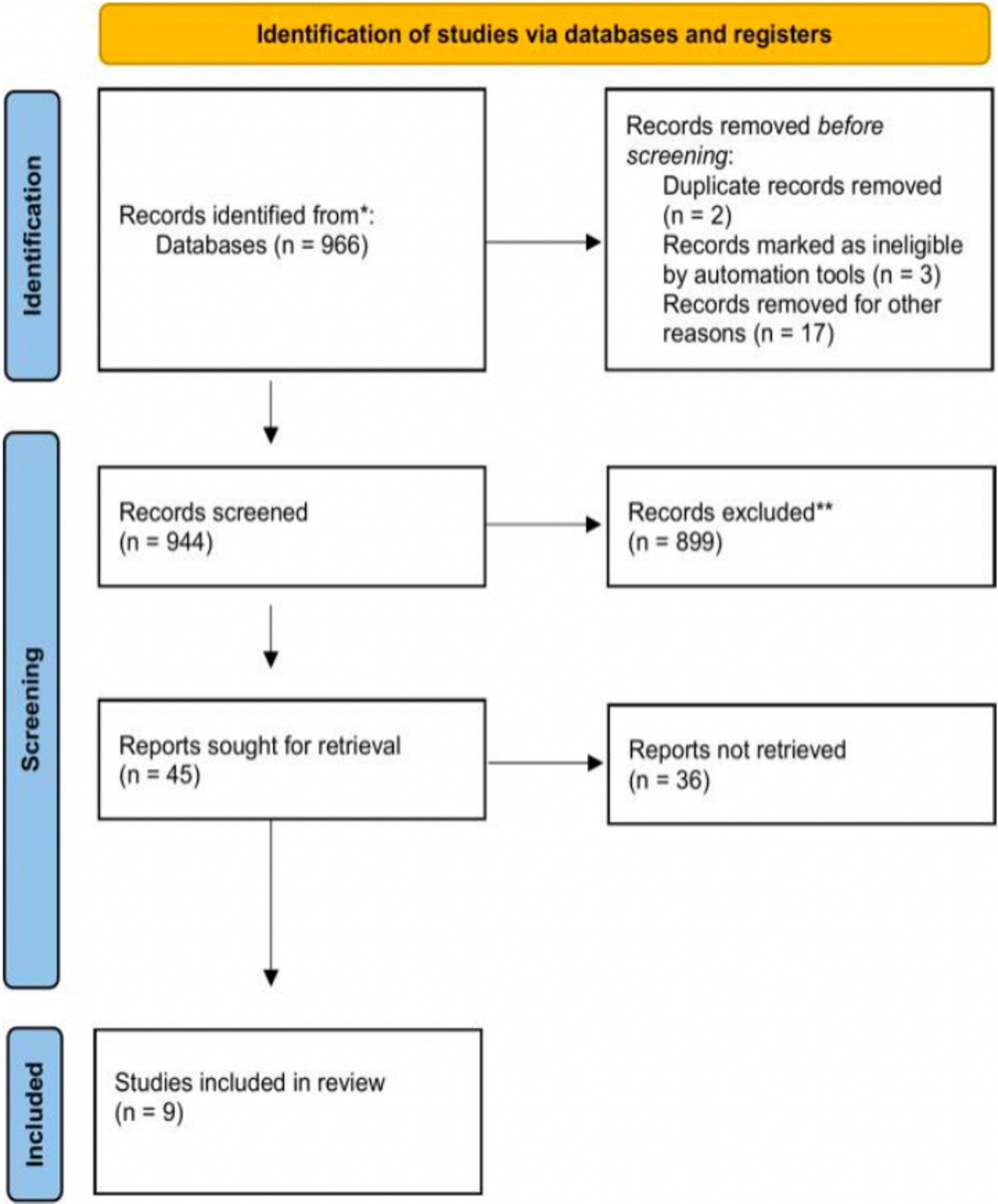
PRISMA flowchart summarizing the study identification, screening, retrieval, and inclusion process.

This umbrella review included nine articles: three systematic reviews and six systematic reviews with meta-analyses (Fig. 1). The general characteristics and epidemiological data of the included reviews are summarized in Table 1.

The citation matrix identified 69 unique primary studies across the nine included reviews, with 121 total study occurrences. The corrected covered area was 9.4 %, indicating moderate overlap among the included reviews. Accordingly, cumulative study and patient counts were interpreted as descriptive characteristics of the review corpus rather than as independent pooled datasets. AMSTAR 2 ratings varied across the included reviews and are reported in Table S2. Overall confidence in the review-level evidence was limited by incomplete reporting of review protocols, variable assessment of publication bias, incomplete discussion of heterogeneity, and possible overlap among primary studies. Across these reviews, 121 primary-study occurrences and 17 708 PJI case entries were reported. However, because these figures are derived from the included reviews and because overlap between primary studies was moderate, they should be interpreted as descriptive characteristics of the review corpus rather than as independent cumulative totals. For this reason, the findings of the present umbrella review are reported primarily at the review level. The mean percentage of CN-PJI across reviews was 33.6 %. At the review level, the reported CN-PJI point estimates were highly variable (11.0 %–63.6 %), with a mean of 33.6 % (SD 16.7), a median of 32.5 %, and an IQR of 23.5 %–38.7 %, underscoring substantial between-review heterogeneity likely driven by differences in pre-analytical conditions, case mixes, microbiological workflows, and diagnostic definitions. Illustratively, large quantitative reviews reported CN proportions of 38.7 % (30 studies; 4207 PJIs) (Li et al., 2023) and 32.5 % (11 studies; 1747 PJIs) (Lai et al., 2024), whereas another meta-analysis reported 11 % (8 studies; 3342 PJIs) (Reisener and Perka, 2018), highlighting how case mix and laboratory protocols can shift estimates. Given the likely overlap among primary studies across the included reviews, we did not consider it to be methodologically appropriate to interpret aggregated raw counts across reviews as a formal pooled prevalence estimate. When risk factors for culture negativity were explicitly quantified, prior antibiotic exposure before sampling emerged as a prominent factor (reported in the 53 %–64 % range in the reviews that provided percentages), consistently with the concept that antimicrobial pretreatment reduces culture yield. Regarding joint distribution, most reviews pooled hip and knee data without stratifying by CN; in the subset where this was available, CN cases were more frequently reported in the knee ( 323/504 , 64.1 %) than in the hip ( 181/504 , 35.9 %). Finally, the diagnostic criteria used to define CN-PJI primarily relied on the MSIS ( 77/121 ), followed by the IDSA ( 15/121 ) and the ICM ( 5/121 ), with smaller contributions from other criteria. This represents an important source of heterogeneity, because CN-PJI is not defined by culture negativity alone but also by the coexistence of negative cultures and diagnostic evidence of infection. Therefore, different diagnostic thresholds may affect both the number of cases classified as PJI and the proportion subsequently categorized as culture-negative. Mean or median follow-up durations for CN-PJI also varied across reviews, ranging from 0.25 to 10 years. Most studies reported follow-up periods of 2–8 years. Molecular diagnostic approaches, treatment strategies, and clinical outcomes are summarized in Table 2.

Within the extracted evidence base, molecular testing in CN-PJI was reported in five out of nine reviews, with a clear predominance of NGS-based approaches: metagenomic NGS (mNGS/shotgun) was mentioned in four out of five molecular-enabled reviews, PCR-based assays were mentioned in one out of five, 16S rRNA sequencing and/or whole-genome sequencing were mentioned in one out of five, and targeted NGS (tNGS) was mentioned in one out of five studies. Across these reviews, specimen acquisition was rarely limited to a single matrix: synovial fluid, periprosthetic tissue, and sonication fluid were all recurrently evaluated, and multiple specimen types (e.g., synovial ± sonication ± tissue) were explicitly considered in all five studies, with biofilm being mentioned in one out of the five, highlighting that molecular yield is typically interpreted in the context of sampling strategy rather than as a “one-sample” paradigm.

When the molecular detection rate (i.e., organism identification among CN-PJI) was reported numerically, estimates were clustered in the mid-range but remained broad: one review reported 43.9 % ( 43/98 ) pathogen detection with shotgun metagenomics (Kalbian et al., 2020) while also citing upper estimates of ∼ 81.8 % for NGS and up to ∼ 90 % for synovial PCR in selected datasets. Other pooled summaries reported overall detection rates of 52.5 %–54.2 % using molecular techniques, with an extreme range of 9 %–100 % across studies, consistently with substantial between-study heterogeneity driven by specimen type, pre-analytic variables, and assay methodology (Wang et al., 2025; Li et al., 2019; Tan et al., 2022; Tang et al., 2022). Diagnostic accuracy metrics were reported more consistently than raw detection rates: across meta-analytic estimates, pooled sensitivity ranged from 0.81 to 0.93 (median 0.84, mean 0.86), and pooled specificity ranged from 0.92 to 0.97 (median 0.94, mean 0.94). In a direct comparison of platforms, mNGS had a sensitivity of 89 % and a specificity of 92 %, whereas tNGS had a sensitivity of 84 % and a specificity of 97 % (Wang et al., 2025). Several reviews consistently reported other metrics, such as true and false negatives (Wang et al., 2025; Li et al., 2023; Tan et al., 2022; Tang et al., 2022), with an overall false-negative proportion of 21.2 % (FN / [TN + FN]). Method-specific extraction from the comparative synthesis yielded FN proportions of 21.7 % for mNGS and 17.1 % for tNGS. Finally, post-molecular pathogen attribution was inconsistently reported and explicitly detailed in only one review (Kalbian et al., 2020). That synthesis reported a notable prevalence of fungal CN-PJI ( ∼ 46 %, predominantly Candida spp.) and detected fastidious or atypical organisms (e.g., mycobacteria, *Cutibacterium acnes*, Brucella, *Coxiella burnetii*) (Kalbian et al., 2020), supporting the concept that “culture-negative” status often reflects microbiological blindspots rather than the absence of infection.

### 3.1 Treatment strategies in CN-PJI

Across the included reviews, treatment strategies for CN-PJI were reported in five out of nine reviews (Table 2). Among these, DAIR was discussed in all five, two-stage exchange was discussed in four out of five, and one-stage exchange was discussed in two out of five, reflecting a broad but non-uniform surgical approach. In the three reviews that provided extractable procedure counts (Lai et al., 2024; Li et al., 2023; Reisener and Perka, 2018), the pooled distribution favored staged revision: two-stage exchange accounted for 1588/2336 cases (68.0 %), DAIR accounted for 508/2336 (21.7 %), and one-stage exchange accounted for 240/2336 (10.3 %). However, differences in case selection and reporting across reviews preclude reliable inference regarding chronicity as a determinant of culture negativity. Antibiotic regimens were described in six out of nine reviews but were often incompletely reported, with dosing and duration frequently omitted or unavailable (NR/NA). Most regimens were empiric, reflecting the lack of culture guidance. Vancomycin and cephalosporins were among the most mentioned agents, generally used to provide broad Gram-positive coverage with additional Gram-negative activity. When treatment pathways were specified, systemic therapy after revision was often reported as approximately 6 weeks, particularly in two-stage protocols, and antibiotic-loaded spacers were commonly used in staged reconstruction. However, because antibiotic selection, dosing, duration, spacer composition, and related stewardship variables were inconsistently described, formal comparison of regimen effectiveness was not possible.

### 3.2 Clinical outcomes

Clinical outcomes for CN-PJI were reported numerically in six reviews (Table 2), but definitions varied (infection control, failure, infection-free survival), limiting direct comparability. In the largest extractable dataset, Li et al. (2023) reported an overall CN-PJI failure rate of 19.0 % ( 309/1630 ), with a 95 % CI of 17.1 %–20.9 %. Within the same synthesis, stratified by surgical approach, failure was lowest after one-stage exchange, although the cohort size was small. In the study by Lai et al. (2024), the pooled infection control rate was 79.2 % in 567 CN cases ( ≈ 449/567 controlled), with an approximate 95 % CI of 75.7 %–82.3 % (assuming the denominator equals the reported CN total). Other reviews reported outcomes primarily as ranges. Reisener and Perka (2018) summarized treatment success at ∼ 85 %–95 % and infection-free survival after two-stage exchange at 67 %–94 % (up to ∼ 95 % at 5 years), while narrative syntheses (Kalbian et al., 2020; Elhence et al., 2025) repeatedly reported two-stage success ranges of ∼ 70 %–100 %, emphasizing high variability across cohorts and protocols.

**Table 1:** Characteristics and epidemiological data of the included reviews. ( n denotes numbers, SR denotes systematic review, MA denotes meta-analysis, PJI denotes periprosthetic joint infection, CN denotes culture-negative, CP denotes culture-positive, MSIS denotes Musculoskeletal Infection Society, and NR denotes not reported)

Reference	Design	Studies ( n )	PJI ( n )	CN ( n )	CN/PJI	CN criteria
Kalbian et al. (2020)	SR	11	NR	NR	23.5 % (5 %–42 %)	MSIS
Lai et al. (2024)	SR and MA	11	1747	567	32.5 % (9.9 %–73.3 %)	MSIS
Li et al. (2023)	SR and MA	30	4207	1630	38.7 %	Mixed
Li et al. (2019)	SR and MA	12	1965	1251	63.6 %	NR
Wang et al. (2025)	SR and MA	23	2992	1597	53.4 %	NR
Reisener and Perka (2018)	SR and MA	8	3342	NR	11 %	MSIS
Yoon et al. (2017)	SR	7	NR	NR	9 %–42 %	MSIS
Tan et al. (2022)	SR and MA	10	NR	NR	NR	NR
Tang et al. (2022)	SR	9	NR	NR	NR	NR

**Table 2:** Molecular diagnostic approaches, treatment strategies, and clinical outcomes reported across the included reviews. ( n denotes numbers, SR denotes systematic review, MA denotes meta-analysis, PJI denotes periprosthetic joint infection, CN denotes culture-negative, CP denotes culture-positive, MSIS denotes Musculoskeletal Infection Society, PCR denotes polymerase chain reaction, NGS denotes next-generation sequencing, and NR denotes not reported).

Reference	Molecular	Test	Specimens	Yield/accuracy	Outcomes
Kalbian et al. (2020)	Yes	PCR, NGS	Variable	Shotgun mNGS positivity 43.9 % ( 43/98 )	High success reported after two-stage revision
Lai et al. (2024)	No	–	–	–	Infection control 79.2 %; empiric antibiotics linked to adverse events
Li et al. (2023)	No	–	–	–	Failure 19.0 % ( 309/1630 ); DAIR worse than exchange
Li et al. (2019)	Yes	Sequencing	Sonication/tissue/ synovial	Pooled Se 0.84; Sp 0.93	Diagnostic-focused
Wang et al. (2025)	Yes	mNGS vs tNGS	Synovial/tissue/ sonication	mNGS Se 0.89 Sp 0.92; tNGS Se 0.84 Sp 0.97	Diagnostic-focused
Reisener and Perka (2018)	No	–	–	–	Success 85 %–95 %; infection-free survival 67 %–94 %
Yoon et al. (2017)	Partial	16S PCR	–	–	Two-stage revision + 4–6 weeks antibiotics: success 70 %–100 %
Tan et al. (2022)	Yes	Shotgun mNGS	Sonication/synovial/ mixed	Yield 54.2 %; pooled Se 0.93 Sp 0.95	–
Tang et al. (2022)	Yes	NGS/met agenomic	Synovial/tissue/ sonication	Yield 52.5 % (9 %–100 %); Se ∼ 0.81	–

## 4 Discussion

This umbrella review found that the reported prevalence of CN-PJI varies widely across published systematic reviews and meta-analyses, reflecting substantial heterogeneity in patient selection, diagnostic criteria, microbiological workflows, and pre-analytical factors. Prior antibiotic exposure before sampling was repeatedly identified as a likely contributor to culture negativity. At the same time, the current evidence base is limited by inconsistent reporting, variable outcome definitions, and probable overlap of primary studies across reviews. The present findings should therefore be interpreted primarily as a review-level overview of the literature rather than as a basis for new pooled estimates of prevalence, treatment effect, or prognosis. In this context, the main value of this umbrella review is to highlight the breadth of heterogeneity in CN-PJI research and the methodological weaknesses that limit direct comparison across reviews. The variability observed across the included reviews is likely explained by a combination of clinical, methodological, and laboratory-related factors. Differences in diagnostic criteria, including MSIS, IDSA, ICM, and other definitions, may have influenced both the number of cases classified as PJI and the proportion considered to be CN. In addition, culture yield may have been affected by pre-analytical and microbiological factors such as prior antibiotic exposure (Wouthuyzen-Bakker et al., 2017), the number and type of samples collected, specimen transport, culture media, the use of sonication or blood culture bottles, and incubation duration. Heterogeneity was also introduced by the use of different specimen matrices, including synovial fluid, periprosthetic tissue, and sonication fluid, as well as by different molecular platforms, such as PCR-based assays, metagenomic NGS, and targeted NGS. Therefore, the differences observed in prevalence, diagnostic performance, treatment strategies, and outcomes should be interpreted not only as clinical variation but also as the result of heterogeneous diagnostic definitions and laboratory workflows.

Our findings are based on the available literature, specifically nine systematic reviews and meta-analyses. There is a pressing need for additional research to fully understand the impact of CN-PJI in arthroplasty. Recent consensus initiatives have also addressed several aspects of CN-PJI diagnosis and management. These documents were not part of the evidence synthesized in the present umbrella review and should therefore be interpreted as external contextual material rather than as review-derived findings. In this external consensus context, the ICM emphasized the importance of obtaining multiple periarticular tissue samples along with synovial fluid (Hoffman et al., 2025) and recommended prolonged incubation of culture specimens for at least 14 d to improve recovery of slow-growing organisms, with extension up to 4 weeks when atypical pathogens such as mycobacteria are suspected (Karim et al., 2025; Tsai et al., 2025). The ICM also proposed a dedicated diagnostic algorithm for patients with CN-PJI and supported the use of molecular techniques as adjunctive tools for pathogen identification in this setting (Tsai et al., 2025; Hansen et al., 2025). Within the evidence synthesized in the present umbrella review, molecular diagnostics were increasingly represented in recent reviews addressing CN-PJI or PJI diagnosis, particularly NGS-based approaches (Li et al., 2019; Tan et al., 2022; Tang et al., 2022; Kullar et al., 2023; Longo et al., 2024; Indelli et al., 2023; Elhence et al., 2025). Across the included diagnostic reviews, molecular diagnostics reported sensitivities of up to 93 % and specificities of up to 97 %. As with standard culture, multiple specimen types (synovial fluid, sonication fluid, periprosthetic tissue) have been evaluated for molecular testing (Wang et al., 2025; Li et al., 2023; Lai et al., 2024; Reisener and Perka, 2018; Kalbian et al., 2020; Yoon et al., 2017; Li et al., 2019; Tan et al., 2022; Tang et al., 2022) to improve sensitivity and specificity. Historically, empiric broad-spectrum antibiotics have often been used for CN-PJI because microbiological guidance has been lacking. Molecular diagnostics may help identify candidate organisms in selected cases and may therefore contribute to more informed antimicrobial decision-making; however, molecular findings alone do not replace phenotypic susceptibility testing, and their impact on truly targeted therapy remains insufficiently defined in the current review-level evidence (Kullar et al., 2023). From a practical perspective, the main diagnostic message is that culture yield should be optimized before an infection is labeled as culture-negative. When clinically feasible, samples should be obtained before antibiotic administration, and diagnostic work-up should include multiple tissue specimens and synovial fluid, appropriate specimen transport and culture media, prolonged incubation, and adjunctive techniques such as blood culture bottles or sonication when available. Molecular diagnostics, including PCR- and NGS-based approaches (Ghirardelli et al., 2024; Indelli et al., 2021; Schoenmakers et al., 2023) may help identify organisms in selected culture-negative cases, but they should be interpreted as adjunctive tools rather than replacements for conventional culture or phenotypic susceptibility testing. Their results should be evaluated in the context of prior antibiotic exposure, specimen type, contamination risk, assay platform, reporting thresholds, and the overall clinical picture.

The current review also analyzed antibiotic use for treating CN-PJIs: the aminoglycoside–vancomycin and cephalosporin–vancomycin combinations were the most commonly used in spacers when a two-stage exchange procedure was selected. We also reviewed the recommended antibiotic treatment between stages. Often, a combination of broad-spectrum antibiotics covering Gram-negative and Gram-positive organisms was used. However, there was again a lack of a consistent, standardized antibiotic regimen. The studies analyzed did not determine when coverage for fungal organisms or atypical pathogens was necessary. In addition, we could not determine from the data whether a 2-week antibiotic holiday improved outcomes in patients undergoing two-stage exchange. Accordingly, these aspects should be interpreted with caution, and the present umbrella review cannot support firm conclusions regarding the optimal antimicrobial strategy for CN-PJI. The recent ICM recommendations, based on emerging data, suggested that antibiotic holidays have no role in these patients and that continuous therapy appears to yield better outcomes, particularly for immunocompromised patients (Elhence et al., 2025). Data on the duration and mode of antimicrobial administration were also scarce. Due to a lack of culture guidance, many studies have reported that 6 weeks of systemic antibiotic treatment and subsequent prolonged oral therapy are necessary (Osmon et al., 2013; Lai et al., 2024; Kalbian et al., 2020; Yoon et al., 2017). The timing of reimplantation has also been controversial among patients undergoing two-stage exchange (Elhence et al., 2025); this issue was particularly pertinent in patients with CN-PJI. This umbrella review has several limitations. First, the included evidence is derived from nine systematic reviews and meta-analyses with moderate overlap among primary studies, as shown by the citation matrix and CCA analysis. Therefore, the reported totals of primary-study occurrences and PJI case entries should not be interpreted as unique cumulative datasets. This overlap supports our decision not to re-pool prevalence, diagnostic performance, treatment, or outcome data across reviews. Second, substantial heterogeneity was observed across reviews in diagnostic definitions, microbiological methods, specimen processing, treatment strategies, and outcome reporting. Third, several clinically relevant variables, including antibiotic selection, dosing, duration, spacer composition, and interval treatment strategies, were incompletely reported. Finally, although overlap among primary studies was formally assessed using a citation matrix and CCA, the presence of moderate overlap further supports the interpretation of the present umbrella review as a descriptive synthesis of review-level evidence rather than as a quantitative pooled estimate of the CN-PJI literature.

## 5 Conclusion

Culture-negative PJI remains a clinically important and heterogeneous entity. Across available systematic reviews and meta-analyses, reported prevalence, diagnostic performance of adjunctive molecular methods, treatment strategies, and outcomes vary substantially. Molecular diagnostics appear to be promising as adjunctive tools in selected cases, but their interpretation requires caution, and they do not resolve all uncertainties related to CN-PJI. Reported treatment outcomes appear to be clinically relevant, but differences in outcome definitions, surgical strategies, antimicrobial protocols, and follow-up duration limit direct comparison across reviews. Given the methodological limitations of the current evidence base, particularly heterogeneity, incomplete reporting, and moderate overlap among primary studies, future high-quality primary studies and rigorously conducted systematic reviews are needed before firm clinical recommendations can be made.

## Supplement

10.5194/jbji-11-479-2026-supplement The supplement related to this article is available online at https://doi.org/10.5194/jbji-11-479-2026-supplement.

## Data Availability

No code was generated or used for this study. The datasets used and/or analyzed during the current study are available from the corresponding author upon reasonable request.
