# Supplementary material for: Culture-negative periprosthetic joint infection: an umbrella review of prevalence, diagnostic strategies, treatment, and methodological limitations
Source: J Bone Jt Infect. 2026 Aug 3;11(4):479–88. doi: 10.5194/jbji-11-479-2026 (PMC13479751; doi:10.5194/jbji-11-479-2026)
Supplement: Supplementary file 1 [file jbji-11-479-2026-supplement.pdf]

Supplement of J. Bone Joint Infect., 11, 479–488, 2026
https://doi.org/10.5194/jbji-11-479-2026-supplement
© Author(s) 2026. CC BY 4.0 License.

Supplement of

Culture-negative periprosthetic joint infection: an umbrella review of prevalence, diagnostic strategies, treatment, and methodological limitations

Guido Bocchino et al.

Correspondence to: Pier Francesco Indelli (indellipf@gmail.com)

The copyright of individual parts of the supplement might differ from the article licence.

Database
MEDLINE via PubMed
Embase
Cochrane Database of Systematic Reviews
Web of Science

Additional search

Complete search strategy
("periprosthetic joint infection"[Title/Abstract] OR "prosthetic joint infection"[Title/Abstract] OR PJI[Title/Abstract] OR "arthroplasty infection"[Title/Abstract] OR "prosthesis-related infection"[Title/Abstract]) AND ("culture-negative"[Title/Abstract] OR "culture negative"[Title/Abstract] OR "negative culture"[Title/Abstract] OR "negative cultures"[Title/Abstract]) AND ("systematic review"[Title/Abstract] OR "meta-analysis"[Title/Abstract] OR "meta analysis"[Title/Abstract] OR review[Publication Type] OR meta-analysis[Publication Type])
('periprosthetic joint infection':ti,ab OR 'prosthetic joint infection':ti,ab OR pji:ti,ab OR 'arthroplasty infection':ti,ab OR 'prosthesis related infection':ti,ab) AND ('culture negative':ti,ab OR 'culture-negative':ti,ab OR 'negative culture':ti,ab OR 'negative cultures':ti,ab) AND ('systematic review':ti,ab OR 'meta-analysis':ti,ab OR 'meta analysis':ti,ab)
("periprosthetic joint infection" OR "prosthetic joint infection" OR PJI OR "arthroplasty infection" OR "prosthesis-related infection") AND ("culture-negative" OR "culture negative" OR "negative culture" OR "negative cultures")
TS=("periprosthetic joint infection" OR "prosthetic joint infection" OR PJI OR "arthroplasty infection" OR "prosthesis-related infection") AND TS=("culture-negative" OR "culture negative" OR "negative culture" OR "negative cultures") AND TS=("systematic review" OR "meta-analysis" OR "meta analysis" OR review)

Reference lists of all included systematic reviews and meta-analyses were manually screened to identify additional eligible reviews not retrieved through the electronic database search.

Metric	Value
Number of included reviews (c)	9
Total primary-study occurrences (N	121
Unique primary studies (r)	69
Corrected covered area (CCA)	0,094
CCA (%)	9,4%
Interpretation	Moderate overlap

Formula / note
c = 9 umbrella review articles
Sum of all 1s in citation matrix / total study entries across reviews
Number of unique primary-study rows after deduplication
(N-r)/(r*c-r)
CCA as percentage
0–5% slight; 6–10% moderate; 11–15% high; >15% very high

Primary study	Primary study note
Berbari 2007	
Dora 2008	
Kobayashi 2008	
Bejon 2010	
Malekzadeh 2010	
Mortazavi 2011	
Gomez 2012	
Huang 2012	
Marin 2012	
Sorli 2012	
Choi 2013	
Rak 2013	
Suda 2013	
Bemer 2014	
Nelson 2014	
Parvizi 2014	Parvizi et al. 2014, Culture-negative periprosthetic joint i
Cha 2015	
Kim 2015a	Kim et al. 2015, J Orthop (comparison of culture-positive
Kim 2015b	Kim et al. 2015, Arch Orthop Trauma Surg (outcome of i
Triantafyllopoulos 2015	
Bereza 2016	
Tan 2016	
Akgun 2017	
Li 2017	
Ibrahim 2018	
Ivy 2018	
Kang 2018	
Larsen 2018	
Santoso 2018	
Tan 2018	
Tarabichi 2018a	Tarabichi et al. 2018, JBJS Am / NGS diagnostic study
Tarabichi 2018b	Tarabichi et al. 2018, second sequencing cohort as liste
Thoendel 2018	
Wang 2018	
Greenfield 2019	Greenfield et al. (as listed in Lai et al. 2024 Table I; publ
Huang 2019	
Ji 2019	
Xu 2019	
Zhang 2019	
Bongers 2020	
Cai 2020	
Fang 2020	
Huang 2020	
Ji 2020	
Theil 2020	
Wang 2020	
Flurin 2021	
He 2021	
Kildow 2021	
Mulpur 2021	
Razii 2021	
Tirumala 2021	

Watanabe 2021	
Yin 2021	
Yu 2021	
Azad 2022	
Flurin 2022	
van Eck 2022	
Veerman 2022	
Xu 2022	
Hao 2023	
Hong 2023	
Huang 2023	
Li 2023a	Li et al. 2023, NGS diagnostic primary study included in
Mei 2023	
Yu 2023	
Zhang 2023	
Shi 2024	
Tan 2024	

Kalbian 2020	Lai 2024	Li 2023	Li 2019	Wang 2025
1	0	0	0	0
0	0	0	1	0
0	0	0	1	0
1	0	0	0	0
1	1	1	0	0
0	0	1	0	0
0	0	0	1	0
1	1	1	0	0
0	0	0	1	0
0	0	1	0	0
1	1	1	0	0
0	0	0	1	0
0	0	0	1	0
0	0	0	1	0
0	0	1	0	0
1	0	0	0	0
0	0	1	0	0
1	1	1	0	0
1	0	1	0	0
0	0	1	0	0
0	0	1	0	0
0	0	1	0	0
0	0	1	0	0
1	1	1	0	0
1	1	1	0	0
0	0	0	1	1
0	0	1	0	0
0	0	0	1	0
0	1	1	0	0
1	0	0	0	0
0	0	0	1	1
0	0	0	1	0
0	0	0	1	1
0	1	1	0	0
0	1	0	0	0
0	0	0	0	0
0	0	1	0	0
0	0	1	0	0
0	0	0	0	1
0	0	1	0	0
0	0	0	0	1
0	0	0	0	1
0	0	0	0	1
0	1	1	0	0
0	0	1	0	0
0	0	0	0	1
0	0	0	0	1
0	0	0	0	1
0	0	0	0	1
0	0	1	0	0
0	0	1	0	0
0	1	1	0	0

0	0	1	0	0
0	0	0	0	1
0	0	0	0	0
0	0	0	0	1
0	0	0	0	1
0	0	1	0	0
0	0	1	0	0
0	0	1	0	0
0	0	0	0	1
0	0	0	0	1
0	0	0	0	1
0	0	0	0	1
0	0	0	0	1
0	0	0	0	1
0	0	0	0	1
0	0	0	0	1
0	0	0	0	1

Reisener 2018	Yoon 2017	Tan 2022	Tang 2022	Row total
1	1	0	0	3
0	0	0	0	1
0	0	0	0	1
0	1	0	0	2
1	1	0	0	5
0	0	0	0	1
0	0	0	0	1
1	1	0	0	5
0	0	0	0	1
0	0	0	0	1
1	1	0	0	5
0	0	0	0	1
0	0	0	0	1
0	0	0	0	1
0	0	0	0	1
0	0	0	0	1
0	0	0	0	1
1	1	0	0	5
1	1	0	0	4
0	0	0	0	1
0	0	0	0	1
0	0	0	0	1
0	0	0	0	1
1	0	0	0	4
1	0	0	0	4
0	0	1	1	4
0	0	0	0	1
0	0	0	0	1
0	0	0	0	2
0	0	0	0	1
0	0	0	1	3
0	0	0	0	1
0	0	1	1	4
0	0	0	0	2
0	0	0	0	1
0	0	1	0	1
0	0	0	0	1
0	0	0	0	1
0	0	1	0	2
0	0	0	0	1
0	0	1	1	3
0	0	1	1	3
0	0	1	1	3
0	0	0	0	2
0	0	0	0	1
0	0	1	1	3
0	0	0	0	1
0	0	1	0	2
0	0	0	1	2
0	0	0	0	1
0	0	0	0	1
0	0	0	0	2

0	0	0	0	1
0	0	0	1	2
0	0	1	0	1
0	0	0	0	1
0	0	0	0	1
0	0	0	0	1
0	0	0	0	1
0	0	0	0	1
0	0	0	0	1
0	0	0	0	1
0	0	0	0	1
0	0	0	0	1
0	0	0	0	1
0	0	0	0	1
0	0	0	0	1
0	0	0	0	1
0	0	0	0	1

Review	Primary study
Kalbian 2020	Ibrahim 2018
Kalbian 2020	Kim 2015a
Kalbian 2020	Li 2017
Kalbian 2020	Malekzadeh 2010
Kalbian 2020	Berbari 2007
Kalbian 2020	Choi 2013
Kalbian 2020	Huang 2012
Kalbian 2020	Bejon 2010
Kalbian 2020	Tan 2018
Kalbian 2020	Parvizi 2014
Kalbian 2020	Kim 2015b
Lai 2024	Greenfield 2019
Lai 2024	Ji 2020
Lai 2024	Tirumala 2021
Lai 2024	Ibrahim 2018
Lai 2024	Santoso 2018
Lai 2024	Wang 2018
Lai 2024	Li 2017
Lai 2024	Kim 2015a
Lai 2024	Choi 2013
Lai 2024	Huang 2012
Lai 2024	Malekzadeh 2010
Li 2023	van Eck 2022
Li 2023	Veerman 2022
Li 2023	Xu 2022
Li 2023	Watanabe 2021
Li 2023	Tirumala 2021
Li 2023	Mulpur 2021
Li 2023	Razii 2021
Li 2023	Theil 2020
Li 2023	Bongers 2020
Li 2023	Ji 2020
Li 2023	Ji 2019
Li 2023	Xu 2019
Li 2023	Wang 2018
Li 2023	Kang 2018
Li 2023	Ibrahim 2018
Li 2023	Santoso 2018
Li 2023	Akgun 2017
Li 2023	Li 2017
Li 2023	Bereza 2016
Li 2023	Tan 2016
Li 2023	Cha 2015
Li 2023	Triantafyllopoulos 2015
Li 2023	Kim 2015b
Li 2023	Kim 2015a
Li 2023	Nelson 2014
Li 2023	Choi 2013
Li 2023	Huang 2012
Li 2023	Sorli 2012
Li 2023	Mortazavi 2011
Li 2023	Malekzadeh 2010

Li 2019	Dora 2008
Li 2019	Kobayashi 2008
Li 2019	Gomez 2012
Li 2019	Marin 2012
Li 2019	Suda 2013
Li 2019	Rak 2013
Li 2019	Bemer 2014
Li 2019	Larsen 2018
Li 2019	Tarabichi 2018a
Li 2019	Thoendel 2018
Li 2019	Ivy 2018
Li 2019	Tarabichi 2018b
Wang 2025	Ivy 2018
Wang 2025	Azad 2022
Wang 2025	Cai 2020
Wang 2025	Fang 2020
Wang 2025	Flurin 2021
Wang 2025	Flurin 2022
Wang 2025	Hao 2023
Wang 2025	He 2021
Wang 2025	Huang 2020
Wang 2025	Huang 2023
Wang 2025	Kildow 2021
Wang 2025	Li 2023a
Wang 2025	Tan 2024
Wang 2025	Thoendel 2018
Wang 2025	Wang 2020
Wang 2025	Yin 2021
Wang 2025	Yu 2023
Wang 2025	Zhang 2019
Wang 2025	Hong 2023
Wang 2025	Mei 2023
Wang 2025	Shi 2024
Wang 2025	Tarabichi 2018a
Wang 2025	Zhang 2023
Reisener 2018	Li 2017
Reisener 2018	Choi 2013
Reisener 2018	Ibrahim 2018
Reisener 2018	Huang 2012
Reisener 2018	Berbari 2007
Reisener 2018	Malekzadeh 2010
Reisener 2018	Kim 2015a
Reisener 2018	Kim 2015b
Yoon 2017	Berbari 2007
Yoon 2017	Bejon 2010
Yoon 2017	Malekzadeh 2010
Yoon 2017	Huang 2012
Yoon 2017	Choi 2013
Yoon 2017	Kim 2015a
Yoon 2017	Kim 2015b
Tan 2022	Thoendel 2018
Tan 2022	Ivy 2018
Tan 2022	Zhang 2019

Tan 2022	Huang 2019
Tan 2022	Cai 2020
Tan 2022	Wang 2020
Tan 2022	Huang 2020
Tan 2022	Fang 2020
Tan 2022	He 2021
Tan 2022	Yu 2021
Tang 2022	Cai 2020
Tang 2022	Fang 2020
Tang 2022	Huang 2020
Tang 2022	Ivy 2018
Tang 2022	Kildow 2021
Tang 2022	Tarabichi 2018a
Tang 2022	Thoendel 2018
Tang 2022	Wang 2020
Tang 2022	Yin 2021

Primary study note
Kim et al. 2015, J Orthop (comparison of culture-positive and culture-negative infected TKA
Parvizi et al. 2014, Culture-negative periprosthetic joint infection
Kim et al. 2015, Arch Orthop Trauma Surg (outcome of infected TKA: culture-positive vs cu
Greenfield et al. (as listed in Lai et al. 2024 Table I; publication year not fully shown in extra
Kim et al. 2015, J Orthop (comparison of culture-positive and culture-negative infected TKA
Kim et al. 2015, Arch Orthop Trauma Surg (outcome of infected TKA: culture-positive vs cu
Kim et al. 2015, J Orthop (comparison of culture-positive and culture-negative infected TKA

Tarabichi et al. 2018, JBJS Am / NGS diagnostic study

Tarabichi et al. 2018, second sequencing cohort as listed separately in Li et al. 2019 table

Li et al. 2023, NGS diagnostic primary study included in Wang et al. 2025

Tarabichi et al. 2018, JBJS Am / NGS diagnostic study

Kim et al. 2015, J Orthop (comparison of culture-positive and culture-negative infected TKA
Kim et al. 2015, Arch Orthop Trauma Surg (outcome of infected TKA: culture-positive vs cu

Kim et al. 2015, J Orthop (comparison of culture-positive and culture-negative infected TKA
Kim et al. 2015, Arch Orthop Trauma Surg (outcome of infected TKA: culture-positive vs cu

Tarabichi et al. 2018, JBJS Am / NGS diagnostic study

Primary study	Number of reviews
Choi 2013	5
Huang 2012	5
Kim 2015a	5
Malekzadeh 2010	5
Ibrahim 2018	4
Ivy 2018	4
Kim 2015b	4
Li 2017	4
Thoendel 2018	4
Berbari 2007	3
Cai 2020	3
Fang 2020	3
Huang 2020	3
Tarabichi 2018a	3
Wang 2020	3
Bejon 2010	2
He 2021	2
Ji 2020	2
Kildow 2021	2
Santoso 2018	2
Tirumala 2021	2
Wang 2018	2
Yin 2021	2
Zhang 2019	2

Review	Design
Kalbian et al. (2020)	Systematic review
Lai et al. (2024)	Systematic review and meta-analysis
Li et al. (2023)	Systematic review and meta-analysis
Li et al. (2019)	Systematic review and meta-analysis
Wang et al. (2025)	Systematic review and meta-analysis
Reisener and Perka (2018)	Systematic review and meta-analysis
Yoon et al. (2017)	Systematic review
Tan et al. (2022)	Systematic review and meta-analysis
Tang et al. (2022)	Systematic review

AMSTAR 2: A Measurement Tool to Assess Systematic Reviews 2; CN-PJI next-generation sequencing.

AMSTAR 2 overall confidence rating
Low
Low
Low
Low
Low
Low
Critically low
Low
Critically low

: culture-negative periprosthetic joint infection

Main methodological limitations
No quantitative meta-analysis; limited formal exploration of heterogeneity; incomplete reporting of treatment and outcome details; possible overlap with later reviews.
Heterogeneity in surgical strategies and outcome definitions; limited standardization of antibiotic regimens and follow-up; possible overlap with previous reviews.
Heterogeneity in included populations, surgical strategies, and outcome definitions; limited granular reporting of microbiological protocols; possible overlap with previous reviews.
Diagnostic-focused review; heterogeneity in sequencing platforms, specimen types, and reference standards; limited direct applicability to CN-PJI treatment outcomes.
Diagnostic-focused review; heterogeneity in mNGS and tNGS platforms, specimen matrices, and diagnostic thresholds; limited treatment and clinical outcome data.
Limited number of included studies; heterogeneity in diagnostic criteria, treatment protocols, and follow-up duration; possible overlap with later reviews.
Narrative synthesis without quantitative pooling; limited number of included studies; incomplete reporting of diagnostic and treatment protocols across primary studies; limited formal assessment of heterogeneity.
Diagnostic-focused review; heterogeneity in mNGS methods, specimen types, and reference standards; limited data on treatment strategies and clinical outcomes.
Diagnostic-focused review; small number of included studies; heterogeneity in NGS methods, specimen types, and diagnostic definitions; limited clinical outcome data and no quantitative pooling.

; mNGS: metagenomic next-generation sequencing; NGS: next-generation sequencing; tNGS: targeted
